# The Cross-Talk between Myeloid and Mesenchymal Stem Cells of Human Bone Marrow Represents a Biomarker of Aging That Regulates Immune Response and Bone Reabsorption

**DOI:** 10.3390/cells11010001

**Published:** 2021-12-21

**Authors:** Maria Elisa Perico, Tommaso Maluta, Giamaica Conti, Antonio Vella, Lisa Provezza, Tiziana Cestari, Giulia De Cao, Lydia Segalla, Cristina Tecchio, Fabio Benedetti, Francesco Santini, Vincenzo Bronte, Bruno Magnan, Andrea Sbarbati, Dunia Ramarli

**Affiliations:** 1 Section of Immunology, Department of Medicine, University of Verona, Policlinico GB Rossi, Piazzale L.A. Scuro 10, 37134 Verona, Italy; antonio.vella@univr.it (A.V.); lisa.provezza@gmail.com (L.P.); tiziana.cestari@univr.it (T.C.); vincenzo.bronte@univr.it (V.B.); 2 Orthopedic and Traumatology Clinic, Department of Surgery, University of Verona, 37134 Verona, Italy; tommasomaluta@yahoo.it (T.M.); giudc@hotmail.it (G.D.C.); bruno.magnan@univr.it (B.M.); 3 Section of Anatomy and Histology, Department of Neuroscience, Biomedicine and Movement Science, University of Verona, 37134 Verona, Italy; giamaica.conti@univr.it (G.C.); lydia.segalla@univr.it (L.S.); andrea.sbarbati@univr.it (A.S.); 4 Section of Hematology and Bone Marrow Transplant Unit, Department of Medicine, University of Verona, 37134 Verona, Italy; cristina.tecchio@univr.it (C.T.); fabio.benedetti@univr.it (F.B.); 5 Section of Cardio Surgery, Department of Surgery, University of Verona, 37134 Verona, Italy; francesco.santini@unige.it; 6 Section of Immunology, Azienda Ospedaliera Universitaria Integrata, 37134 Verona, Italy; dunia.ramarli@gmail.com

**Keywords:** aging, bone marrow, adipogenesis, suppression, IL-6

## Abstract

One of the mechanisms that characterizes the aging process of different organs is the accumulation of fat. Different authors have demonstrated that adipose tissue replaces the loss of other cell types, deriving from mesenchymal cells. During aging, there is substitution or trans-differentiation of mesenchymal cells with other cells having the same embryological origin. Newly formed adipocytes were also observed in the trabecular matrix of elderly people’s bones, associated with myeloid cells. In this study, we have investigated the relationship between immature myeloid-derived suppressor cells (I-MDSCs) and mesenchymal stem cells (MSCs) in bone marrow (BM) samples harvested from 57 patients subjected to different orthopedic surgeries. Patients aged from 18 to 92 years were considered in order to compare the cellular composition of bone marrow of young and elderly people, considered a biomarker of immunity, inflammation, and bone preservation. The I-MDSC percentage was stable during aging, but in elderly people, it was possible to observe a strong basal immunosuppression of autologous and heterologous T cells’ proliferation. We hypothesized that this pattern observed in elders depends on the progressive accumulation in the BM of activating stimuli, including cell–cell contact, or the production of different cytokines and proteins that induce the differentiation of bone marrow mesenchymal stem cells in adipocytes. The collected data provided underline the importance of specific biomarkers of aging that promote a reduction in immune response and incremented inflammatory pathways, leading to bone reabsorption in elderly people.

## 1. Introduction

A common hallmark of the aging of primary lymphoid organs in humans is the progressive accumulation of adipose tissue [1]. In the thymus, true epithelial spaces are sheltered from adipose tissue by columnar epithelial borders [2,3], whereas in the bone marrow, the myeloid and the adipocyte components live in direct contact in an ever-remodeling microenvironment. In fact, the young, sound bone trabecular structure filled with myeloid cells is gradually replaced during aging by a rarefied, often demineralized trabecular net where low numbers of myeloid cells are surrounded by an overwhelming adipocyte population [4,5,6].

The generation of fat in bone marrow is presumed to be similar to adipogenesis in other tissues. A correlation between adipose accumulation in the bone marrow and decreases in bone mass has been reported in pathological conditions associated with bone loss, such as aging [7,8,9]. As both osteoblasts and adipocytes originate from MSCs [10], a large number of extracellular and intracellular signals and transcription factors balance adipogenesis and osteoblastogenesis [11,12]. The central components of these networks are CCAAT/enhancer-binding protein beta (C/EBPβ), peroxisome proliferator-activated receptor γ (PPAR-γ), and Wnt [13], transcriptional factors commonly differentiated by adipocytes and osteocytes [14]. An imbalanced adipogenic/osteogenic ratio leads to the commitment of MSCs to one lineage. Directly linked to C/EBPβ activation is the phosphorylation of p38MAPK [15]. The inhibition of p38MAPK activity in 3T3-L1 fibroblasts blocks their differentiation in adipocytes by decreasing the phosphorylation of C/EBPβ and of PPAR-γ [16], required for terminal adipogenesis, as well as the expression of specific adipocyte genes such as adiponectin, GLUT4, and leptin. A recent study demonstrated a correlation between the expression of IL-6R and MB-MSCs’ adipogenesis differentiation, accompanied by the same trend of p38 phosphorylation, thus suggesting that the IL-6/IL-6R pathway plays a significant role in tissue regeneration and cell differentiation [17].

A balance between hematopoietic stem cell (HSC) quiescence and HSC cycling ensures the normal turnover of blood cells, protects stem cells from premature exhaustion, and sustains efficient hematopoiesis. Although the cellularity of the bone marrow decreases with age, the number of HSCs has been shown to increase with age [18,19]. However, old human HSCs are less quiescent compared to young human HSCs, thus limiting their ability to effectively self-renew and reconstitute the hematopoietic system [20]. Mesenchymal stem cells (MSCs) are critical components of the bone marrow niche, and recent evidence suggests that senescence decreases MSCs’ capacity to promote HSC quiescence, reducing the frequency of CD34^+^CD38-. The decrease in HSCs’ quiescence is linked to the increase in senescence-associated IL-6 production by both senescent and adult MSCs [21].

Myeloid-derived suppressor cells (MDSCs) are a heterogeneous population of immature myeloid precursor cells that function as negative regulators of immune responses by inhibiting T cell proliferation, IFN-gamma production, and CTL induction and have been reported to play important roles in the development of microbial inflammation and infection [22,23,24]. Circulating MDSCs have been found to increase during chronic viral infection, malignancies, and aging [25,26,27,28], mostly in frail or community-dwelling elders where they may worsen an already poor response to infection and vaccination [29,30].

Of note, previous reports have shown that MDSCs’ suppressive activity can be triggered by IL-6, IL-1 beta, TNF-alpha, and S100A9 protein, all produced by adipose tissues and elevated in elders [31,32,33].

All the evidence described above indicates that the disarray of the bone marrow architectural structure deeply affects the functional interplay between HSCs, MSCs, MDSCs, extracellular matrix (ECM) proteins, and the variety of soluble interactors constituting the BM microenvironment. As a result, MSCs skew their differentiation potential toward adipocytes rather than balancing the generation of osteocytes, chondrocytes, and adipocytes, and HSCs reduce their self-renewing potential while increasing myeloid differentiation and decreasing the generation of common lymphoid progenitors [5,19,20].

To better describe these relationships, in this study, we have investigated the inducing effect of I-MDCS/BM-MSC cross-talk on the suppressive activity of myeloid cells and on adipogenesis in human BM during aging.

## 2. Materials and Methods

### 2.1. Patients and Healthy Donors

This study was approved by the Institutional Ethics Committee for Clinical Studies (approval no. CE2129 and CE1727). Written informed consent was obtained from each patient and protected according to the ethics guidelines.

The study was conducted on a group of 57 subjects (31 females and 26 males), enrolled with an open scheme lasting 36 months (Table 1). Healthy donors and patients were arbitrarily divided into three groups: young people (*n* = 18; ages 18–35 years; mean age 26 ± 4.8), adults (*n* = 17; ages 36–65 years; mean age 53 ± 9.6), and elders (*n* = 22; ages 66–92 years; mean age 80 ± 8.6). The young and elder groups were homogeneously composed of bone marrow (BM) donors or patients who had undergone femur fracture. The adults group was instead composed of six BM donors, four patients who had undergone cardio surgery, and seven with a femur fracture, due to the difficulty of recruiting a homogeneous number of volunteers aged 36–65 years. BM aspirates and peripheral blood were obtained from all patients. None of them had history of cancer, HIV or HCV infection, thalassemia, or corticosteroid treatment in the last six months before enrolment or showed obesity (mean BMI 22 ± 3.3 kg/m^2^).

### 2.2. Bone Marrow Hematocytometric and Flow Cytometric Analysis

Fresh BM samples were diluted 1:5 with PBS and analyzed with a hematocytometer to count white blood cells (WBCs), red blood cells (RBCs), and the percentage of neutrophils. Immunophenotyping was then performed using two calibrated mixtures of conjugated mAbs: the first evaluated the myeloid compartment, containing the mAbs anti-HLA-DR-FITC (clone G46-6, BD Biosciences, San Jose, CA, USA), anti-CD11b-PE (clone ICRF44, BD Bioscience), anti-CD45-PerCP (clone 2D1, BD Bioscience, San Jose, CA, USA), anti-CD14-PE-Cy7 (clone M5E2, BD Bioscience), anti-CD33-APC (clone WM53, BD Bioscience), and anti-CD16^−^ APC-Cy7 (clone 3G8, BD Bioscience), and the second evaluated the lymphoid compartment, composed of the Abs anti-CD56-PE (clone MY31, BD Bioscience), anti-CD8-PE-Cy7 (clone RPA-T8, BD Bioscience), anti-CD3-APC (clone SK7, BD), anti-CD19-APC-Cy7 (clone SJ25C1, BD Bioscience), and anti-CD4-FITC (clone L200, BD Bioscience). Cells were stained for 30 min at 4 °C in the dark in PBS containing 2% FCS buffer, washed, and analyzed using a Navios Flow Cytometer (Beckman Coulter Inc. Brea, CA, USA).

### 2.3. Plasma Recovery and Cytokine Detection

BM and peripheral blood plasma were recovered by centrifugation, filtered, and stored at −80 °C until use. ELISA for human cytokines IL-6, IL-1 beta, TNF-alpha (Thermo-Fisher, Waltham, MA, USA), and Calgranulin S100A9 (Cloud-Clone Corp, Katy, TX, USA) was performed according to the manufacturer’s instructions.

### 2.4. Immunomagnetic Sorting

Fresh BM samples were cleared of tissue debris, depleted of RBCs by ammonium chloride lysis, and left to adhere to the plastic for 1 h at 37 °C in DMEM (Lonza Group Ltd., Basel, Switzerland) at 5% FCS. Non-adherent cells were recovered and depleted of CD3^+^, CD19^+^, CD56^+^, and CD14^+^ cells (Lin^+^ fraction) with a cocktail of immune magnetic beads following the manufacturer’s instructions (Miltenyi Biotec, Bergisch Gladbach, Germany). The Lin^−^ cells were either used in the induction of suppression assays or further depleted of CD11b^+^ cells by a second run of sorting with CD11b immune magnetic beads (Miltenyi Biotec) and immediately used in suppression assays. The ≥98% negatively sorted cells were Lin^−^CD16^−^CD11b^−^ as evaluated by flow cytometry after every sorting.

### 2.5. Induction of Suppression Activity and Suppression Assay

Induction of suppression was carried out by culturing Lin^−^ myeloid cells for 4 days in IMDM (Biochrom GmbH, Berlin, Germany) with 10% FCS and 1mM HEPES (Sigma-Aldrich, St. Louis, MO, USA) in the presence of G-CSF and GM-CSF (40 ng/mL each) (Miltenyi Biotec) or rIL-6 (5 ng/mL) or co-cultured at a 20:1 cell ratio with BM-MSCs. If needed, BM-MSCs were cultured on glass disks fitting 24-well plates for 24 h prior to co-cultivation. Lin^−^ cells were gently recovered and subjected to immunomagnetic sorting as previously detailed. Both CD16^−^CD11b^+^ and CD16^−^CD11b^−^ fractions were assayed in suppression assays. Suppression assay was conducted using heterologous or autologous PBMCs previously stained with 5 µM Cell Trace Violet (Invitrogen, Carlsbad, CA, USA) and plated at 10^4^ cells/well in round-bottomed 96-well Costar plates pre-coated with 1 µg/mL of anti-CD3 mAb (clone UCHT1, Thermo-Fisher). The CD16^−^ CD11b^+^ or CD16^−^ CD11b^−^ myeloid cells were co-cultured for 4–5 days at a 2:1, 1:1, or 0.5:1 myeloid/PBMC ratio in final volume of 200 µL of L-Arginine-free IMDM containing 10% FCS, 1 mM HEPES, 150 µm L-Arginine, and 1.5 µg/mL of soluble anti-CD28 mAb (Thermo-Fisher). PBMCs stimulated in the absence of myeloid cells or not stimulated represented the controls. At the end of the experimental time, cells were stained with an anti-CD3-APC mAb (Miltenyi Biotec), and their proliferation was assessed using Cell Trace (Invitrogen) or CFSE (Invitrogen) signals analyzed by Navios Flow Cytometry gating on CD3^+^ lymphocytes (T cells). Suppression was calculated as the ratio between the percentage of proliferating T cells cultured in the absence or presence of myeloid cells (positive control) at the various ratios.

### 2.6. BM-Derived MSC (BM-MSC) Culture, Characterization and Adipogenic Differentiation

Adherent cells obtained from BM samples were cultured in 75cm^3^ flasks in DMEM with 5% FCS for one week and in 10% FCS until they reached confluence. Their MSC origin was established by flow cytometry assessing the co-expression of beta1-integrin (CD29, clone TS2/16, Thermo-Fisher), CD105 (clone 266, BD Bioscience), and CD44 (Beckman Coulter) in the absence of CD45 (BD Bioscience) surface molecules. Differentiation into adipocytes was carried out by soluble inducers or cell–cell contacts. BM-MSCs were plated onto glass disks in a 24-well Costar plate at 5 × 10^2^ cells/well in standard medium and cultured for 24–48 h. Firmly adherent cells were washed and kept in culture in the adipogenic medium (AM) composed of DMEM, 10% FCS, 0.2 mM IBMX (Sigma-Aldrich), 10 µM Rosiglitazone (Sigma-Aldrich), 0.4 µg/mL hydrocortisone, and 5 µg/mL insulin, feeding the cells two times on every 3rd day. Adipogenic medium was substituted by maintenance medium (DMEM, 10% FCS and 5 µg/mL insulin) for 7–10 days, and cells were used for staining or co-culture experiments or as a source of cell lysates. If needed, adipogenic medium was replaced in the same experimental setup by BM plasma (for 24–48 h), used at a final concentration of 20%, or by recombinant IL-6 (Thermo-Scientific), used at 3 ng/mL. For cell–cell contacts, CD16^−^CD11b^−^ cells or CD16^−^CD11b^+^ myeloid cells were admixed at a 20:1 myeloid/BM-MSC ratio; if needed, co-cultures were performed in the presence of 25 μM p38MAPK inhibitors SB202190 (Calbiochem-Novabiochem, San Diego, CA, USA), preincubated for 20 min before the experimental start and present throughout the experimental time. Accumulation of lipid drops ensured the occurrence of adipogenesis. If required, cultures were performed in the presence of 20 μg/mL of the recombinant humanized anti-IL-6R monoclonal antibody tocilizumab (RoActemra, Roche, Basel, Switzerland).

### 2.7. Red Oil Staining

Cells were washed in PBS, fixed in 2% glutaraldehyde–2% formaldehyde–4% sucrose in H_2_O for 2 h at room temperature, carefully washed in PBS, and stained for 20 min at room temperature with filtered Oil red-O staining solution (0.5 g Oil red-O powder in 60% isopropanol) (Red Oil from Bio-Optica, Milano, Italy). Cells were washed with ddH_2_O. Images were acquired under a microscopeOlympus BX51 (Olympus, Tokyo, Japan) for analysis of lipid droplets.

### 2.8. Immunoblotting (IB)

IB was performed according to standard techniques with cell lysates prepared as previously described and proteins quantified by Biorad protein assay reagent (Hercules, CA, USA). Samples were analyzed by SDS-PAGE under reducing conditions and electroblotted to nitrocellulose membranes (Biorad). Membranes were probed with the following monoclonal or polyclonal Abs: phospho-p38 MAPK, Perilipin (Cell Signaling Technology, Danvers, MA, USA), C/EBPβ (Santa Cruz Biotechnology Inc., Dallas, TX, USA), phospho-STAT3^Y705^ (Abcam, Cambridge, UK), and Actin (Sigma-Aldrich). HP-conjugated anti-rabbit and anti-mouse IgG were from Millipore (Temecula, CA, USA). Signals were detected by ECL (Lite AbLot Extend or Turbo Western blot detection kits) (EuroClone, Milano, Italy).

### 2.9. BM-MSC Cloning

Cloning of BM-MSCs was performed in duplicate by limiting dilution at 1 cell/well in a final volume of 150 µL in 96-well Costar plates pre-coated for 48 h/overnight with DMEM containing 10% FCS. Three days after plating, the standard medium was replaced by adipogenic medium in one of the two replica plates. Media were changed every third day. At day 10 from plating, media were removed, and cells were fixed with 10% ethanol in H_2_O and 0.001% crystal violet in H_2_O. Cells were counted with an optical microscope at 40× magnification. If needed, cells were subsequently stained with red oil and analyzed with the optical microscope.

### 2.10. Statistical Analysis

All the data are presented as the mean ± SD. Non-parametric results were analyzed by two-tailed Mann–Whitney–Wilcoxon tests, considering significance at *p* ≤ 0.05. Parametric results were analyzed by the two-tailed Student’s t-test considering significance at *p* ≤ 0.05.

## 3. Results

### 3.1. Cell Subset and Cytokine Levels Are Remodeled during Aging of the Bone Marrow

**Figure 1:**
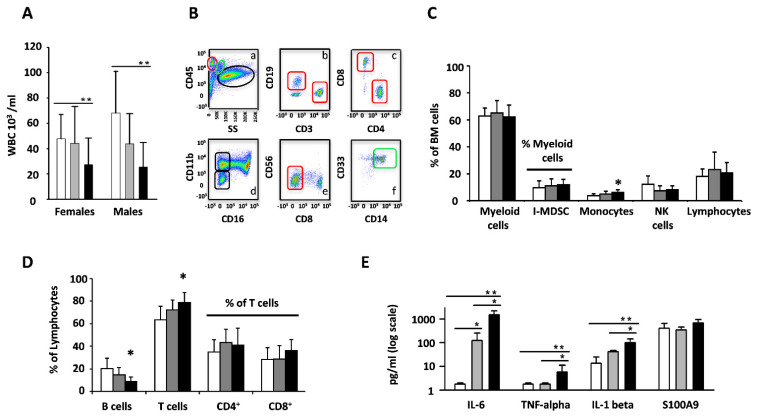
Cells and cytokines of human BM during aging. White, gray, and black colors in all panels respectively indicate young people, adults, and elders. (**A**). Absolute number of WBCs in female and male young people, adults, and elders. (**B**). Representative plot of multicolor flow cytometry showing lymphoid (red), monocytes (green), and myeloid (black) cells of BM mapped on an initial CD45^+^/Side Scatter (SS) plot (panel **a**) and subsequently identified as CD45^high^ CD19^−^ CD3^+^ T lymphocytes (T cells) and CD45^high^ CD3^−^ CD19^+^ B lymphocytes (B cells) (panel **b**); CD45^high^ CD19^−^ CD3^+^ CD8^−^ CD4^+^ T lymphocytes (CD4^+^ T cells) and CD45^high^ CD19^−^ CD3^+^ CD4^−^ CD8^+^ T lymphocytes (CD8^+^ T cells) (panel **c**); CD45^high^ CD56^+^ NK cells (panel **e**); CD45^high^ CD3^−^ CD33^+^ CD14^+^ monocytes (panel **f**); CD45^low^ CD3^−^ CD33^+^ CD16^−^ CD11b^+^ myeloid cells (CD11b^+^ cells) and CD45^low^ CD3^−^ CD33^+^ CD16^−^ CD11b^−^ myeloid cells (I-MDSCs) (panel **d**). (**C**,**D**). Mean values ± SD of the percentages of the populations in (**B**) in young people, adults, and elders. (**E**) Mean values ± SD of IL-6, TNF-alpha, IL-1 beta and S100A9 in BM of young people, adults, and elders. * *p* < 0.05, ** *p* < 0.01.

In patients, a reduction in white blood cells (WBCs) with aging was registered, as shown in Figure 1A. The decrement was not correlated with the gender of the patients. A further detailed multicolor flow cytometry analysis (Figure 1B) demonstrated that WBCs, although decreasing in absolute numbers, did not modify the relative percentage of myeloid cells, the immature MDSC (I-MDSC) subset (CD33^+^ CD45^low^CD3^−^CD16^−^CD11b^−^), total lymphocytes, or NK cells (Figure 1C,D). In contrast, B cells almost halved in elders (Figure 1D). The percentage of monocytes and T cells increased slightly but significantly, still maintaining the normal ratio of CD4^+^/CD8^+^ cells (Figure 1D). In contrast, IL-6, IL-1 beta, and TNF-alpha progressively and significantly increased with aging in BM plasma, while S100A9 protein remained unchanged in the same samples. Noteworthily, the IL-6 concentration raised from less than 2 pg/mL in young people to more than 1 ng/mL in elders (Figure 1E). The number of RBCs and the percentage of neutrophils, a marker of traumatic inflammation, were similar in all groups, thus ruling out the likelihood that the increase in cytokines observed during aging depended on the different sites of BM sampling or the traumas (Table S1).

### 3.2. Ex Vivo Suppressive Activity of I-MDSCs Is Primarily Found in Elders

**Figure 2:**
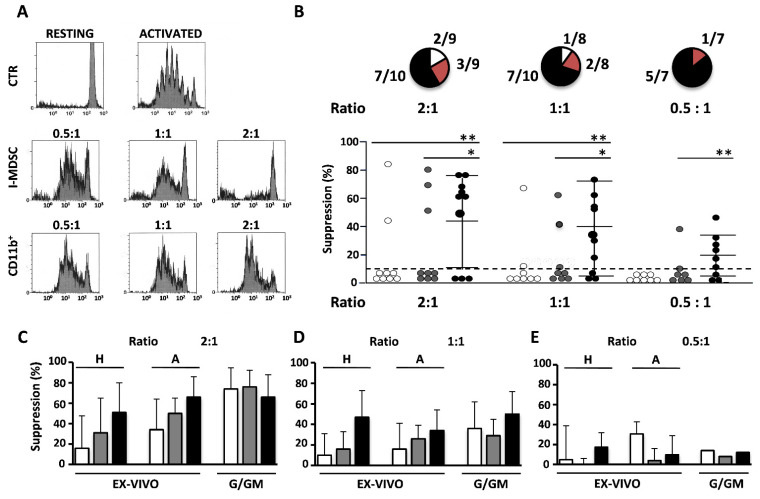
Ex vivo and inducible I-MDSC suppression observed during aging. (**A**). Representative suppression assay analyzed by flow cytometry. Plots refer to CD3-gated T lymphocytes non-stimulated (RESTING), activated in the absence (ACTIVATED) or in the presence of lymphocyte–monocyte-depleted CD33^+^ CD11b^−^ CD16^−^ cells (I-MDSCs) or CD33^+^ CD11b^+^ CD16^−^ (CD11b^+^) cells at the indicated ratios. Mean value of T cell activation was 90 ± 8% SD. (**B**) (upper panel). Number of BM samples containing active I-MDSCs out of the indicated number of elders (black portion), adults (red portion), and young people (white portion). (**B**) (lower panel). Percentage of heterologous ex vivo suppression of I-MDSCs of young people (white dots), adults (grey dots), and elders (black dots) at the indicated ratio. Dot values are the mean of duplicates. Dotted line indicates the 10% of suppression arbitrarily considered negative. (**C**–**E**). Mean value ± SD of the percentages of heterologous (H) and autologous (A) ex vivo suppression (EX VIVO) and of the heterologous G/GM-induced (G/GM) suppression of young people (white bars), adults (grey bars), and elders (black bars) at the indicated I-MDSC/PBMC ratio, respectively of 2:1 (**C**), 1:1 (**D**) and 0.5:1 (**E**). *p*-values as in Figure 1. * *p* < 0.05, ** *p* < 0.01.

The suppression activity of the I-MDSC population was assessed immediately after their isolation (“ex vivo suppression”) or upon treatment with G/GM CSF [25]. As previously shown in Figure 1C, the percentage of I-MDSCs in the myeloid cell compartment was unrelated to aging. To assess whether their suppressive function was instead modified, we tested the ex vivo suppression of I-MDSCs purified (≥ 97%) from BM samples of 9 young people, 9 adults, and 10 elders (Figure 2). Suppression was evaluated by flow cytometry comparing the proliferation of heterologous T cells detected in PBMC stimulated by mAb-mediated T cell receptor/co-receptor engagement in the absence or presence of I-MDSCs at a 2:1, 1:1, or 0.5:1 I-MDSC/PBMC ratio. As shown in a representative assay performed with I-MDSCs from an elder (Figure 2A), the 88% proliferating T cells found in PBMCs stimulated in the absence of I-MDSCs decreased progressively when increasing the number of I-MDSCs (72, 65, and 23% at 2:1, 1:1, and 0.5:1, respectively). Ex vivo suppression was exerted only by I-MDSCs; the CD33^+^CD16^−^CD11b^+^ (CD11b^+^) cells failed to exert a comparable activity. By using this assay, we found (Figure 2B, upper panel) that 70% of elders (black portion) and 33% of adults (red portion) but only 22% of young people (white portion) showed ex vivo suppression of heterologous T cell proliferation at a 2:1 cell/cell ratio. Suppression was still detectable in all elders at a 1:1 cell ratio and in five of them at a 0.5:1 cell/cell ratio. The low number of young people and adults declined rapidly as none of the young people and only one adult showed detectable activity at a 0.5:1 cell/cell ratio. The mean values of each individual are shown in Figure 2B (lower panel) and further detailed in Table S2. We next evaluated whether I-MDSC suppression was inducible or maintained in the various individuals by canonical treatment with G/GM CSF [25] and whether the ex vivo suppression observed equally suppressed the proliferation of heterologous and autologous T cells. The results shown in Figure 2C–E (and Table S2) compare young people’s, adults’, and elders’ I-MDSCs in terms of ex vivo suppression of heterologous (H) and autologous (A) T cell proliferation and G/GM-induced suppression of heterologous T cell proliferation. They clearly demonstrate that the I-MDSCs found in young people (white columns), adults (grey columns), and elders (black columns) could equally suppress heterologous (H) and autologous (A) T cell proliferation cells, thus suggesting that ex vivo suppression observed in vitro may also be exerted in vivo. In addition, young people’s, adults’, and elders’ I-MDSCs could all be efficiently induced to suppress by treatment with G/GM-CSF, thus suggesting that the lack of ex vivo suppression observed in young people and adults depended on the lack of stimuli in the microenvironment rather than on functional immaturity and that, conversely, elders’ I-MDSCs could have been induced in vivo by stimuli accumulating in BM during aging.

### 3.3. BM-MSCs and BM-MSC-Derived Adipocytes Efficiently Induce the Suppressive Activity of I-MDSCs

**Figure 3:**
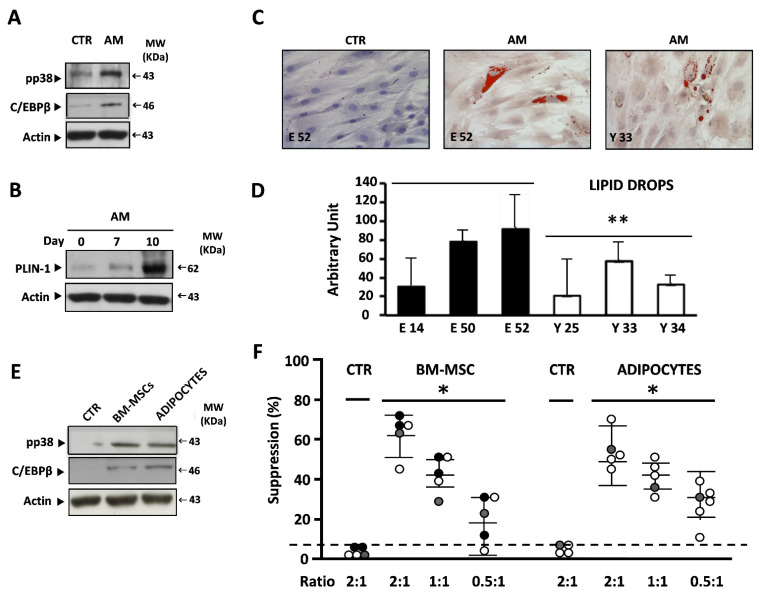
BM-MSCs or BM-MSC-derived adipocytes efficiently induce the suppressive activity of I-MDSCs. (**A**). Representative immunoblotting analysis of p38 MAPK phosphorylation and C/EBPβ expression in BM-MSCs treated with adipogenic medium (AM) on Day 3. β-Actin ensured equal loading in the lanes. Arrows indicate relevant bands in all panels. (**B**). Representative immunoblotting analysis with kinetic (Day 0, Day 7, and Day 10) of PLIN-1 accumulation in BM-MSCs during the treatment with AM. β-Actin ensured equal loading in the lanes. Arrows indicate relevant bands in all panels. (**C**). Representative morphology of BM-MSCs and BM-MSC-derived adipocytes from both young people (Y33) and elders (E52), stained with red oil, after 10 days of treatment with AM. (**D**). Diagrams of pixel quantification of lipid drops content, relative to images in Figure 3C, generated with ImageJ (http://imagej.nih.gov/ij/, 1997–2011, accessed on 21 December 2021), in BM-MSC cultures from young or elder donors, treated with AM. Values are the mean ± SD of area pixels obtained in at least three independent experiments. (**E**). Representative immunoblotting analysis of p38 MAPK phosphorylation and C/EBPβ expression in I-MDSCs co-cultured with either BM-MSCs or BM-MSC-derived adipocytes on Day 4. β-Actin ensured equal loading in the lanes. Arrows indicate relevant bands in all panels. (**F**). Percentage of suppression of the proliferation of heterologous PBMCs by I-MDSCs co-cultured in presence of BM-MSCs (left) or adipocytes derived from BM-MSCs (right). Dots represent the mean of co-cultures performed in duplicate. * *p* < 0.05, ** *p* < 0.01.

To investigate adipocytes’ role as inducers of I-MDSC suppressive activity, we derived BM-MSC cultures from six young people’s and six elders’ BMs. Their MSC origin was ensured by the spindle-like morphology associated with the co-expression of CD105, CD29, and CD44 molecules in the absence of CD45 antigens (Figure S1). As shown in Figure 3A, incubation with adipogenic medium (AM) induced the phosphorylation of p38 MAPK (pp38) and the transcription of C/EBPβ transcription factor, followed by the increasing accumulation of Perilipin 1 (PLIN-1), a peculiar component of lipid drops (Figure 3B) [34,35]. Accordingly, lipid drops were detected by red oil staining in BM-MSC cultures derived from young people or elders (Figure 3C). However, the pixel densitometry of red particles revealed that both the number and the size of lipid drops were three times greater in elders than in young people (Figure 3D).

BM-MSCs or BM-MSC-derived adipocytes (hereon called Adipocytes) derived from young people’s or elders’ BMs were co-cultured for 4 days with Lin^−^ myeloid cells obtained from young people, adults, and elders. I-MDSCs recovered from co-cultures and purified by immunomagnetic sorting (98%) were used in suppression assays. The representative results shown in Figure 3E demonstrate that the contact with either BM-MSCs or adipocytes did activate—in young people’s, adults’, and elders’ I-MDSCs—the p38 MAPK-C/EBPβ pathway, previously reported to regulate the induction of suppressive activity in humans, together with a strong, significant suppression of the proliferation of heterologous PBMCs (Figure 3F).

### 3.4. Myeloid Cells and Soluble Factors Secreted during Co-Culture Induce IL-6 Gene Expression and Adipogenesis in BM-MSCs

**Figure 4:**
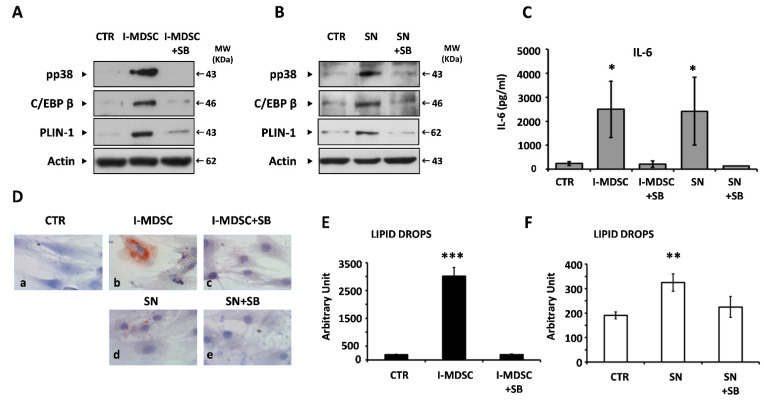
Myeloid cells and soluble factors secreted during co-culture induce IL-6 gene expression and adipogenesis in BM-MSCs. (**A**,**B**). Representative immunoblotting analysis of the phosphorylation of p38 MAPK, the expression of the 47 KDa isoform of C/EBPβ, and PLIN-1 accumulation in BM-MSCs co-cultured with I-MDSCs (**A**) or treated with co-culture supernatants (**B**) in the presence or absence of SB. β-Actin ensured equal loading in the lanes. Arrows indicate relevant bands in all panels. (**C**). IL-6 production of BM-MSCs cultured with the indicated stimuli. Values are the mean ± SD of results obtained in several independent experiments. (**D**). Representative red oil staining of BM-MSCs cultured in standard medium (panel **a**), co-cultured with I-MDSCs in the absence (panel **b**) or presence (panel **c**) of the p38 MAPK inhibitor SB202190 (SB), or treated with BM-MSC/I-MDSC co-culture supernatants in the absence (panel **d**) or presence (panel **e**) of SB. (**E**,**F)**. Diagrams of pixel quantification of lipid drops content relative to images in (**D**), generated with ImageJ (http://imagej.nih.gov/ij/, 1997–2011, accessed on 21 December 2021), in BM-MSC cultures. Values are the mean values ± SD of area pixels obtained in at least three independent experiments. * *p* < 0.05, ** *p* < 0.01, *** *p* < 0.001.

The possibility that myeloid cells could also exert biological activity on BM-MSCs is shown in Figure 4A, in which it can be observed that contact with I-MDSCs induced activation of the p38 MAPK and C/EBPβ pathways and the accumulation of PLIN-1 in BM-MSCs. C/EBPβ was also inducible by the treatment supernatant of co-cultures (Figure 4B). In addition, a great reduction in pp38, C/EBPβ, and PLIN-1 expression was observed in BM-MSCs if the activation of p38 MAPK was prevented by using the specific inhibitor SB202190 (SB) at a concentration of 25 µM (Figure 4A,B). The results summarized in Figure 4C clearly demonstrate that I-MDSC contact or co-culture supernatants induced IL-6 gene expression and a significant increase in the cytokine release in the variously treated cultures. SB treatment almost abrogated the induction exerted by I-MDSC contact or co-culture supernatant. Overall, these results confirmed in our cellular system that the p38 MAPK/C/EBPβ pathways are required for IL-6 gene expression and activated by cellular contacts and soluble factors.

It is well known that the activation of C/EBPβ not only leads to IL-6 gene expression but also drives adipogenesis [11]. To investigate whether these stimuli triggered BM-MSCs to differentiate into mature adipocytes, the BM-MSCs were cultured in a similar experimental setup for four days, washed, kept in maintenance medium for a week, and stained with red oil. As depicted in Figure 4D, the cell contact with I-MDSCs induced a complete and strong differentiation of BM-MSCs (panel a: LD content 190 ± 14) into mature adipocytes (panel b: LD content 3019 ± 299; *p* < 0.0001). The same activity was fully inhibited by SB treatment (panel c: LD content 196 ± 18). Supernatants of BM-MSC/I-MDSC co-cultures significantly induced adipogenesis, although to a lesser extent (panel d: LD content 324 ± 35; *p* < 0.001). Furthermore, in this case, adipogenesis was abrogated by SB treatment (panel e: LD content 224 ± 42). Pixel quantification diagrams related to Figure 4D are reported in Figure 4E,F.

### 3.5. IL-6 Is an Adipogenic Factor in BM-MSCs

**Figure 5:**
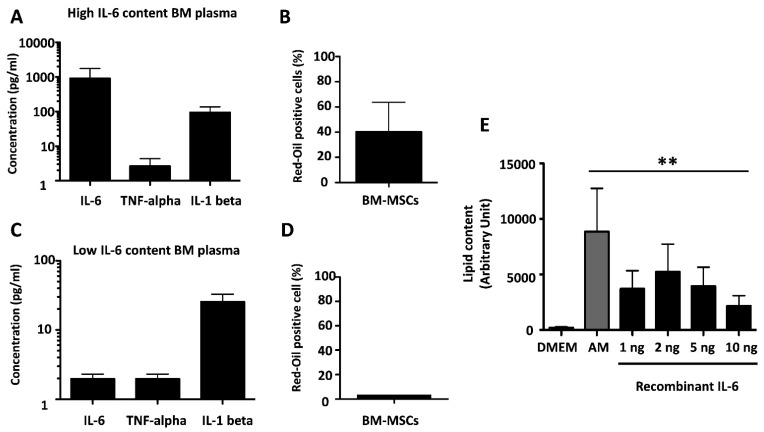
Adipogenesis induced by IL-6-containing BM plasma or by recombinant IL-6. (**A**). IL-6, TNF-alpha, and IL-1 beta concentration in BM plasma assayed in (**B**). (**B**). Percentage of red-oil-positive cells in MSCs treated with four independent “high IL-6 producer” BM plasmas. (**C**). IL-6, TNF-alpha, and IL-1 beta concentration in BM plasma assayed in (**D**). (**D**). Percentage of red-oil-positive cells in MSCs treated with four independent “low IL-6 producers” BM plasmas. (**E**). Lipid content of BM-MSCs upon treatment with recombinant IL-6 at the indicated doses, expressed as mean ± SD of results of pixel densitometry. ** *p* < 0.01.

To investigate whether IL-6 triggered BM-MSCs to differentiate into mature adipocytes, two BM-MSC cultures were treated with eight independent BM plasmas containing similar amounts of IL-1 beta and TNF-alpha but differing in the amounts of IL-6. The four plasmas containing high amounts of IL-6 (>800 pg/mL) induced adipogenesis in two independent MSC cultures as assessed by red oil staining (Figure 5A,B), whereas the plasmas containing low amounts of IL-6 (<5 pg/mL) did not (Figure 5C,D). To confirm that IL-6 could be adipogenic for BM-MSCs, three independent BM-MSC samples were cultured in the presence of the indicated doses of recombinant IL-6 or in AM (as control). As shown in Figure 5E, a dose-dependent induction was observed, as assessed by pixel densitometry of lipid drops. This demonstrated that IL-6 is able to induce full adipogenesis in BM-MSCs, thus making it likely that native IL-6 in BM plasma accounted for their activity.

### 3.6. IL-6 Mediates the Adipogenic Effect of BM Plasma on BM-MSCs

**Figure 6:**
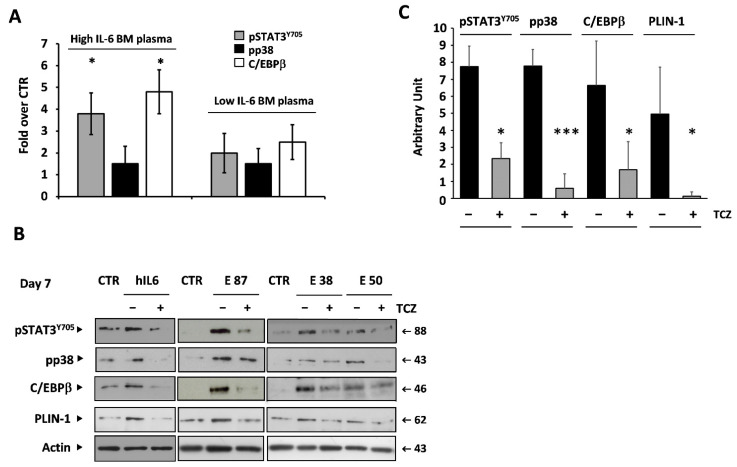
IL-6 mediates the adipogenic effect of elder BM plasma on BM-MSCs, activating the pSTAT3Y705 and C/EBPβ pathways and inducing PLIN-1 accumulation in BM-MSCs. (**A**). Diagrams of mean ± SD of pixel densitometry of Western blot analysis of pSTAT3^Y705^, pp38, and C/EBPβ induction in BM-MSCs treated with BM plasma from elder donors containing a high or low amount of IL-6. Values are calculated as fold increase over control. (**B**). Crude lysates (10 μg) of BM-MSCs treated with recombinant human IL-6 (3 ng/mL) or BM plasma from elder donors (final concentration 20%) in the presence or absence of TCZ (20 μg/mL) immunoblotted with the indicated mAbs. β-Actin ensured equal loading in the lanes. Arrows indicate relevant bands in all panels. (**C**). Pixel densitometry of B. Values are the mean ± SD of area pixels obtained in at least three independent experiments. * *p* < 0.05, *** *p* < 0.001.

The presence of a high amount of IL-6 in the elder BM plasma could then explain its adipogenic effect on BM-MSCs. BM plasma samples from eight independent elder donors containing similar amounts of IL-1 beta and TNF-alpha but differing in the level of IL-6 were compared for the ability to activate, on BM-MSCs, the IL-6-mediated signaling pathway. BM plasma containing a high level of IL-6 (>800 pg/mL) induced a statistically significant increase in both STAT3 phosphorylation at Tyr705 (pSTAT3^Y705^) and C/EBPβ activation in BM-MSCs. The induction of p38 MAPK was similar but not statistically significant (Figure 6A). Moreover, the treatment of BM-MSCs with three independent elder BM plasma samples (E87, E38, E50) containing high amounts of IL-6 or with recombinant human IL-6 (3 ng/mL), as a control, clearly induced a strong activation of p38 MAPK, pSTAT3^Y705^, and C/EBPβ, which was inhibited by tocilizumab (TCZ) [36,37]. We also analyzed the expression of PLIN-1, a protein coating the surface of lipid droplets (LDs), involved in the early stages of the differentiation process of LD formation. Our results show that the treatment of BM-MSCs with BM plasma containing a high amount of IL-6 induced the expression of PLIN-1, which is hampered by treatment with TCZ. The pixel densitometry of the blots shown in Figure 6B is reported in Figure 6C.

### 3.7. BM-MSCs from Elders Are Prone to Adipocyte Differentiation

**Figure 7:**
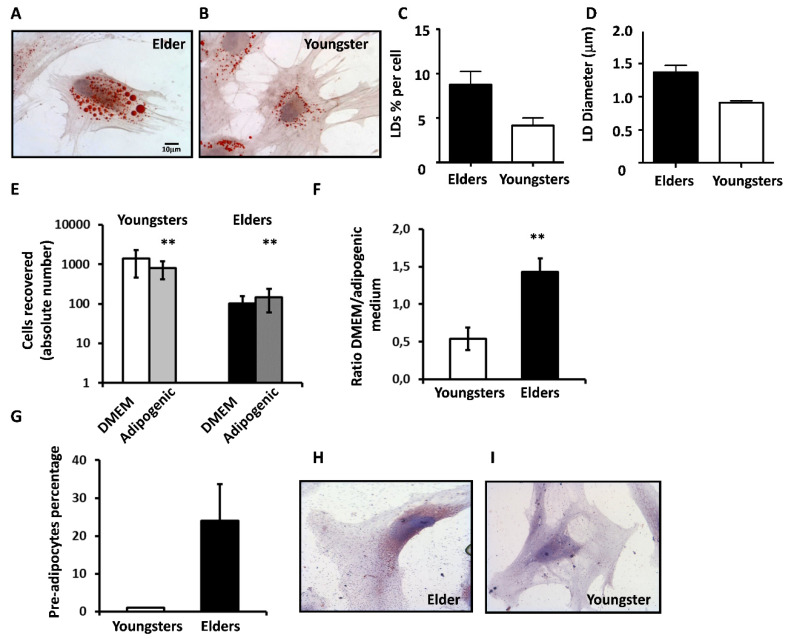
BM-MSCs of elders are prone to adipocyte differentiation. (**A**,**B**). Representative red oil staining of elder (**A**) and young (**B**) BM-MSCs cultured in AM on Day 9. (**C**). Mean values ± SD of the percentage of lipid drops (LDs) content per cell in young (white) and elder (black column) BM-MSCs. (**D**). Mean values ± SD of the LD diameter (μm) in young (white) and elder (black column) BM-MSCs. (**E**). Absolute number of cells recovered from young and elder BM-MSCs, cloned by limiting dilution in DMEM or AM. (**F**). Ratio between the number of recovered cells from both young and elder BM-MSCs grown in AM or in DMEM. (**G**). Percentage of young or elder positive clones stained with red oil. (**H**,**I**). Representative red oil re-staining of all DMEM plates of elder (**H**) and young (**I**) BM-MSCs. In all panels, data are expressed as mean ± SD, ** *p* < 0.01.

Elders’ BM-MSCs (Figure 7A) differentiated faster than young people’s BM-MSCs (Figure 7B), producing a higher number of larger lipid drops (Figure 7C,D).

To investigate whether BM-MSCs from elders were prone to adipocyte differentiation, we compared six cultures from young people and six from elders using a limiting dilution assay performed in parallel in standard DMEM or AM. If considering at least three proximate cells as a “clone”, the cloning efficiency of young people’s BM-MSCs was similar in DMEM and AM (41.3 ± 28.5% and 41 ± 32.5%, respectively), whereas that of elder BM-MSCs was significantly higher in AM than in DMEM (32 ± 13 versus 21 ± 11). The number of cells recovered from all young BM-MSCs in DMEM (1391 ± 936) almost halved in AM (797 ± 377), whereas that for elders, although reduced to one-tenth, raised from 92 ± 54 in DMEM to 124 ± 88 in AM (Figure 7E). This opposite response to AM of young and elder BM-MSCs was even more evident if calculating the ratio between the number of cells grown in DMEM and AM for each culture. As shown in Figure 7F, the mean ratio of the six young people’s BM-MSCs was 0.59 ± 0.15, whereas that of elders reached 1.43 ± 0.18. Moreover, as we observed that clones from elders in DMEM showed small lipid drops at the end of the experimental time, we re-stained all DMEM plates with red oil, finding that 24 ± 9.5 of elder clones (Figure 7G,H), but none of the young ones (Figure 7G,I), showed the reddish staining of pre-adipocytes.

## 4. Discussion

In this study, we have investigated some of the biomolecular interactions taking place in human BM during aging. Our data show that the cross-talk between myeloid and mesenchymal stem cells of human bone marrow induces suppression in I-MDSCs and adipogenesis in BM-MSCs. The study was conducted in a group of 57 volunteers free of infectious, neoplastic, or genetic diseases, ranging from 18 to 92 years of age. We are aware that our number of patients may be not sufficiently large, but the accurate patient enrolment (none of them had history of cancer, HIV or HCV infection, thalassemia, or corticosteroid treatment in the last six months before enrolment or showed obesity) may strengthen the value of our observations.

It is common knowledge that the number of white blood cells in the human BM decreases with aging; this phenomenon was also observed in our volunteers, independent of sex. The analysis of myeloid and lymphoid populations and subpopulations demonstrated that the nearly 50% decrease in WBCs in elders’ BM was quite homogeneously distributed among the cell subpopulations, with the exception of B cells that halved in the elders. In particular, we could not detect age-dependent variations in the percentage of I-MDSCs. Despite the stability of the percentage of I-MDSCs during aging, only those freshly isolated from elders showed an intense and basal (otherwise called spontaneous) suppressive activity on the proliferation of heterologous and, perhaps more importantly, autologous T cells. It can be conceived that the I-MDSC population present in young people was, in any case, too low in number to sustain dilution, whereas this population in elders, being greatly enlarged, was detectable when diluted. It is tempting to speculate that the I-MDSC suppression observed on autologous T cells likely to recirculate in the body may also impair antigen-specific responses in these patients. The finding that both young people’s and elders’ I-MDSCs could be efficiently induced to suppress, upon treatment with G/GM-CSF, suggested that elders’ I-MDSCs had already been induced in vivo. As previously mentioned, the architectural structure of young people’s BM progressively subverts during aging, ending in a microenvironment where the hematopoietic component is overwhelmed by adipocytes and the adipokines that they produce [37,38]. Our finding that BM-MSCs or adipocytes derived from BM-MSCs induced the suppressive activity in I-MDSCs demonstrated that both BM-MSCs and the adipose tissue surrounding myeloid cells may account for the activation of suppression in vivo and, therefore, for the spontaneous suppression that we detected in freshly isolated I-MDSCs. In agreement with our observations, a growing number of studies on the BM niche have demonstrated that both BM-MSCs and BM adipocytes play a key role in the maintenance of the BM microenvironment, respectively acting as regulators of the hematopoietic stem and progenitor cells’ homeostasis and supporting hematopoietic stem cell survival [9,21,39]. Furthermore, our data demonstrated that cell–cell contact between BM-MSCs or BM-MSC-derived adipocytes and I-MDSCs appeared to be strictly required for the induction of suppression.

Moreover, we observed an additional phenomenon resulting from the BM-MSC/I-MDSC interaction. I-MDSCs activated adipogenic differentiation in BM-MSCs starting from the activation of p38 MAPK and the expression of C/EBPβ transcription faction, then proceeding towards the morphological changes of pre-adipocytes and the perinuclear accumulation of lipid drops, which is a peculiar feature of mature adipocytes. Noteworthily, this process was associated with the concomitant induction of gene expression of IL-6. The induction of adipogenesis in BM-MSCs was also inducible by supernatants of BM-MSC/I-MDSC co-cultures.

The putative role of IL-6 in these phenomena was confirmed using an assay of induction of adipogenesis in BM-MSCs performed with increasing doses of recombinant IL-6 that could mediate the adipogenic effect (by activation of p38 MAPK, pSTAT3^Y705^, and C/EBPβ and inducing the expression of PLIN), which in turn was hampered by treatment with the anti-IL-6R monoclonal antibody, tocilizumab. Our data show that adipogenesis in BM-MSCs was also inducible by soluble factors, namely those from “high IL-6 producer” BM plasma. Thus, the presence of high amounts of IL-6 in elder BM plasma could then explain its adipogenic effect on BM-MSCs that we found to be already committed to adipocyte differentiation.

These results demonstrate that the BM-MSC population remodels during the aging of the BM. Young people’s and elders’ BM-MSCs share the same morphology, peculiar phenotype, and molecular pathway regulating the induction of adipocyte differentiation. However, elders’ BM-MSCs decrease in proliferation while increasing in sensitivity to adipogenic induction and commitment to adipocyte differentiation. Interestingly, a recent study [40,41] identified, in murine bones, a stem-cell-like population producing osteogenic and adipogenic lineages, the latter expansion being specifically promoted by aging.

Collectively, our data demonstrate that a detrimental cross-talk occurs in elders’ BM, resulting in the induction of suppression in I-MDSCs and adipogenesis in BM-MSCs, most likely induced or maintained by both cell–cell contacts and IL-6. These results open up new possibilities for defining markers and targets for preventive or therapeutic strategies in aging to eventually regulate immunity, inflammation, and bone resorption in elders [42,43,44].

## Figures and Tables

**Table 1:** Demographic and clinical features of BM donors and patients.

Volunteers	Total Number	Age (Range)	Mean Age ± SD	BM Donors		Cardiac Surgery		Femur Fracture	
				F	M	F	M	F	M
Young people	18	18–35	26 ± 4.8	8	10	0	0	0	0
Adults	17	36–65	53 ± 9.6	4	2	0	4	5	2
Elders	22	66–92	80 ± 8.6	0	0	0	4	14	4
Site of BM sampling				Iliac crest	Iliac crest	Sternum	Sternum	Femur	Femur
Total number	57			12	12	0	8	19	6

## Data Availability

Not applicable.

## Supplementary Materials

The following supporting information can be downloaded at: https://www.mdpi.com/article/10.3390/cells11010001/s1, Table S1: Absolute number (concentration) of WBCs and RBCs and percentage of neutrophils according to age or site of BM sampling. Table S2: Percentage of suppression of heterologous (Hetero) or autologous (Auto) T cell proliferation exerted by young people’s, adults’, or elders’ I-MDSCs either purified freshly (EX VIVO) or upon treatment with G/GM CSF (G/GM induced). Figure S1: Representative BM-MSCs’ characterization. A. Morphology. B. Representative plot of multicolor flow cytometry showing the expression/adhesion cell markers CD44^+^ (glycoprotein), CD29^+^ (β-integrin), and CD105^+^’s (endoglin) expression in the absence of hematological marker CD45.

## Abbreviations

BM	Bone marrow
BM-MSC	Bone marrow mesenchymal stem cell
CD	Cluster of differentiation
C/EBP beta	CAAT/enhancer-binding protein β
DMEM	Dulbecco’s Modified Eagle Medium
ECL	Enhanced chemiluminescence
ECM	Extracellular matrix
ELISA	Enzyme-linked immunosorbent assay
FACS	Fluorescence activated cell sorting
FCS	Fetal calf serum
G-CSF	Granulocyte colony-stimulating factors
GM-CSF	Granulocyte macrophage colony-stimulating factors
HEPES	4-(2-hydroxyethyl)-1-piperazineethanesulfonic acid
HP	Horseradish peroxidase
HSC	Hematopoietic stem cell
IBMX	3-isobutyl-1-methylxanthine
IL	Interleukin
IMDM	Iscove’s Modified Dulbecco’s Medium
Lin	Lineage
mAb	Monoclonal antibody
MDSC	Myeloid-derived suppressor cell
I-MDSC	Immature myeloid-derived suppressor cell
MAPK	Mitogen-activated protein kinase
MSC	Mesenchymal stem cell
PBMC	Peripheral blood mononuclear cell
PBS	Phosphate-buffered saline
PPAR-γ	Peroxisome proliferator-activated receptor-g
RBC	Red blood cell
SDS-PAGE	Sodium dodecyl sulphate polyacrylamide gel electrophoresis
STAT	Signal transducers and activators of transcription
TNF-alpha	Tumor necrosis factor alpha
WBC	White blood cell
