# Supplementary material for: The Cross-Talk between Myeloid and Mesenchymal Stem Cells of Human Bone Marrow Represents a Biomarker of Aging That Regulates Immune Response and Bone Reabsorption
Source: Cells. 2021 Dec 21;11(1):1. doi: 10.3390/cells11010001 (PMC8750773; doi:10.3390/cells11010001)
Supplement: Supplementary file 1 [file cells-11-00001-s001.zip › cells-1517218-supplementary.pdf]

cells

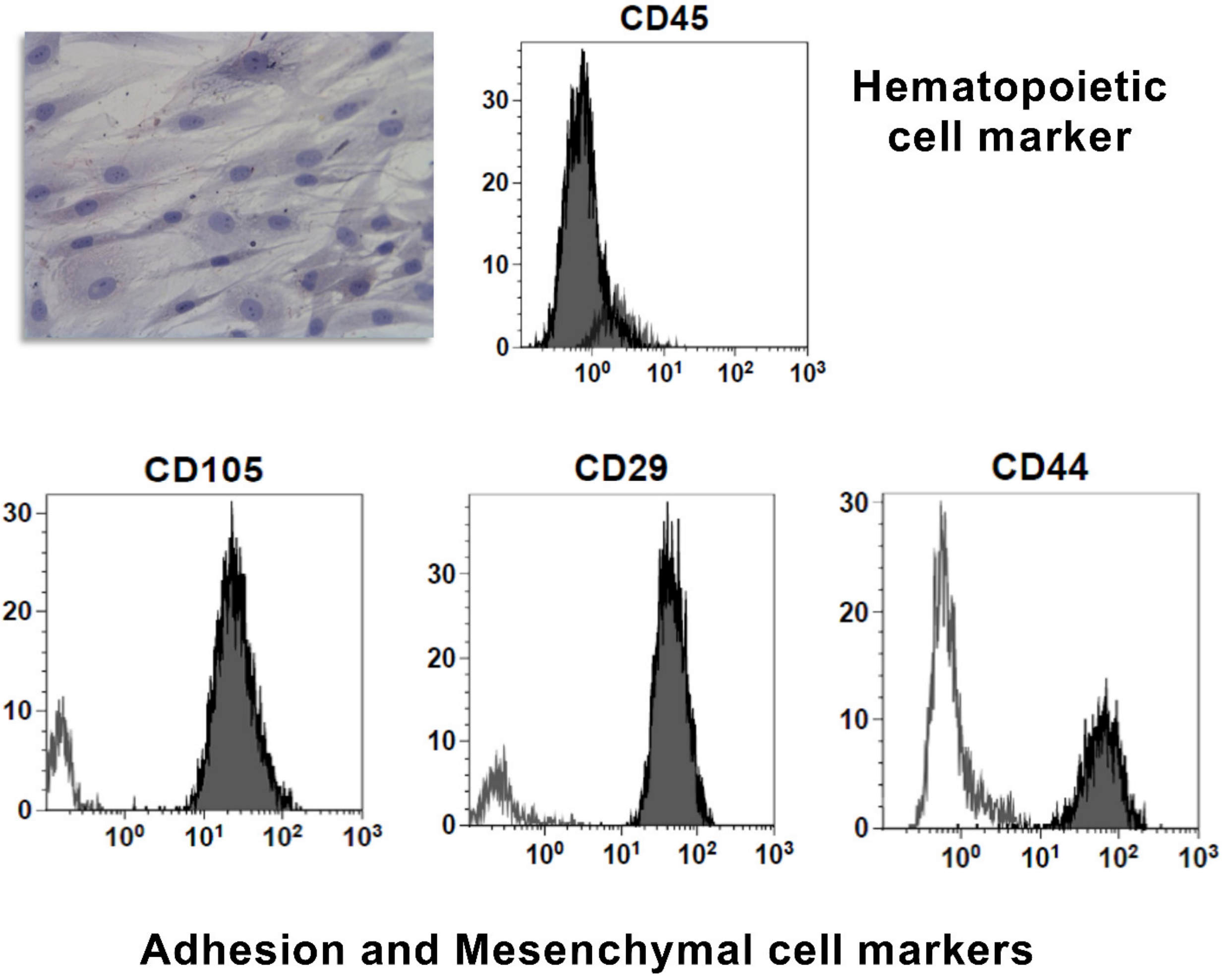

Figure S1. Representative BM-MSCs′ characterization. A. Morphology. B. Representative plot of multicolor flow cytometry showing the expression/adhesion cell markers $CD44^+$ (glycoprotein), $CD29^+$ (β-integrin), and $CD105^+$'s (endoglin) expression in the absence of hematological marker CD45.

Supplementary Table S1. Absolute number (concentration) of WBCs and RBCs and percentage of Neutrophils, according to age or site of BM sampling.

**Volunteers**	**Youngsters**	**Adults**	**Elders**	**Site of BM sampling**		
**Age (range)**	**18-35**	36-65	65-92	Femur	Iliac Crest	Sternum
**WBC (10^3/ml)**	68413 ± 32914	40711 ± 29031*	25532 ± 18047**	31650 ± 220305	52280 ± 26307	18725 ± 6167
**RBC (10^6/ml)**	4.22 ± 1	3.45 ± 2	3.35 ± 1.2	4.29 ± 1	3.45 ± 2	4.7 ± 3
**Neutrophils (%)**	62.45 ± 6	64 ± 12	63 ± 2	62.14 ± 14	51 ± 15	53 ± 16

* $p < 0.05$.

Supplementary Table S2. Percentage of suppression of heterologous (Hetero) or autologous (Auto) T cell proliferation exerted by youngsters or adults or elders I-MDSCs either purified freshly (EX VIVO) or upon treatment with by G/GM CSF (G/GM induced).

**Ratio I-MDSC/PBMC**	**2:1**			**1:1**			**0.5:1**		
**I-MDSC**	EX VIVO		G/GM induced	EX VIVO		G/GM induced	EX VIVO		G/GM induced
**Target**	Hetero	Auto	Hetero	Hetero	Auto	Hetero	Hetero	Auto	Hetero
Youngsters	16 ± 32	34 ± 30	74 ± 21	10 ± 21	16 ± 17	47 ± 26	5 ± 14	0	17 ± 19
Adults	31 ± 34	50 ± 15	75 ± 8	16 ± 25	26 ± 14	34 ± 20	9 ± 15	4 ± 6	10 ± 16
Elders	51 ± 24	66 ± 20	66 ± 22	36 ± 26	29 ± 16	49 ± 20	14 ± 12	8 ± 12	14 ± 19
